# The environmental zero‐point problem in evolutionary reaction norm modeling

**DOI:** 10.1002/ece3.3929

**Published:** 2018-03-25

**Authors:** Rolf Ergon

**Affiliations:** ^1^ University College of Southeast Norway Porsgrunn Norway

**Keywords:** adaptive peak, genetic assimilation, multivariate breeder's equation, perception traits, reaction norms, reference environment, state‐space modeling

## Abstract

There is a potential problem in present quantitative genetics evolutionary modeling based on reaction norms. Such models are state‐space models, where the multivariate breeder's equation in some form is used as the state equation that propagates the population state forward in time. These models use the implicit assumption of a constant reference environment, in many cases set to zero. This zero‐point is often the environment a population is adapted to, that is, where the expected geometric mean fitness is maximized. Such environmental reference values follow from the state of the population system, and they are thus population properties. The environment the population is adapted to, is, in other words, an internal population property, independent of the external environment. It is only when the external environment coincides with the internal reference environment, or vice versa, that the population is adapted to the current environment. This is formally a result of state‐space modeling theory, which is an important theoretical basis for evolutionary modeling. The potential zero‐point problem is present in all types of reaction norm models, parametrized as well as function‐valued, and the problem does not disappear when the reference environment is set to zero. As the environmental reference values are population characteristics, they ought to be modeled as such. Whether such characteristics are evolvable is an open question, but considering the complexity of evolutionary processes, such evolvability cannot be excluded without good arguments. As a straightforward solution, I propose to model the reference values as evolvable mean traits in their own right, in addition to other reaction norm traits. However, solutions based on an evolvable ***G*** matrix are also possible.

## 1 INTRODUCTION

Reaction norms are extensively used in evolutionary modeling of population systems where the individuals have the ability of phenotypic plasticity, that is, where organisms can change their phenotypes in response to changes in the environment (Chevin & Lande, 2015; Ergon & Ergon, 2017; Gavrilets & Scheiner, 1993a,b; Gomulkiewicz & Kirkpatrick, 1992; Lande, 2009, 2014; Schlichting & Pigliucci, 1998). Such models are special cases of state‐space models, with basically three equations. First, an individual reaction norm model describes how a multivariate individual phenotype ***y***_*i*,*t*_ is expressed as a linear or nonlinear function of quantitative traits ***z***_0,*i*,*t*_ and a continuously varying multivariate developmental environment (environmental cue) ***u***_*t*_,

(1) $$y_{i,t}=g\left(z_{0,i,t},u_{t}-u_{\mathrm{ref}}\right).$$

Here, ***z***_0,*i*,*t*_ may be the individual parameter vector as function of time *t* (generations) in a parametrized model of the reaction norm, or alternatively the individual phenotypic values at discrete index environments. Interpolation between index environments results in a function‐valued or infinite‐dimensional individual reaction norm model $y_{i,t}=\gamma \left(u_{t}-u_{\mathrm{ref}}\right)$ (Kingsolver, Gomulkiewicz, & Carter, 2001; Kirkpatrick & Heckman, 1989; Kirkpatrick, Lofsvold, & Bulmer, 1990). The reference environment is often set to ***u***_ref_ = 0 (Gavrilets & Scheiner, 1993a,b; Lande, 2009), but that disguises the problem at hand. Second, the individual fitness function is

(2) $$W_{i,t}=h\left(y_{i,t}-\theta_{t}\right),$$

where **θ**_*t*_ is the vector of phenotypic expression that maximizes fitness in the given generation. Note that in the univariate and linear case, the covariance between *u*_*t*_ and θ_*t*_ determines the mean reaction norm slope in a stationary stochastic environment (Ergon & Ergon, 2017; McNamara, Barta, Klaassen, & Bauer, 2011). Third, the state equation that propagates the mean trait values may under given assumptions be the multivariate breeder's equation (Lande, 1979)

(3) $${\overset{¯}{z}}_{0,t+1}={\overset{¯}{z}}_{0,t}+\frac{1}{{\overset{¯}{W}}_{t}}GP^{-1}\text{cov}\left(W_{i,t},z_{0,i,t}\right).$$

Equation (3) is based on the assumption that the phenotypic traits can be split into two mutually independent and multinormally distributed parts, ***z***_0,*i*,*t*_ = ***x***_0,*i*,*t*_ + ***e***_0,*i*,*t*_, with the covariance matrices $G=E\left[\left(x_{0,i,t}-{\overset{¯}{x}}_{0,t}\right){\left(x_{0,i,t}-{\overset{¯}{x}}_{0,t}\right)}^{T}\right]$ and $E=E\left[\left(e_{0,i,t}-{\overset{¯}{e}}_{0,t}\right){\left(e_{0,i,t}-{\overset{¯}{e}}_{0,t}\right)}^{T}\right]$,respectively. As a consequence also ***z***_0,*i*,*t*_ is multinormally distributed, with the covariance matrix $P=E\left[\left(z_{0,i,t}-{\overset{¯}{z}}_{0,t}\right){\left(z_{0,i,t}-{\overset{¯}{z}}_{0,t}\right)}^{T}\right]$. I will here assume ***P*** and ***G*** to be constant, which is common in theoretical work (e.g., Lande, 2009), although it is unrealistic over longer time periods (Steppan, Phillips, & Houle, 2002). I will assume populations with non‐overlapping generations, where all individuals live in the same time‐varying environment, and make standard assumptions necessary for the multivariate breeder's equation (3) to be valid (Lande, 1979). For analytical purposes, expressions for mean values ${\overset{¯}{y}}_{t}$ and ${\overset{¯}{W}}_{t}$ can be found from equations (1) and (2), but they are not needed for simulations.

The fundamental insight is that the reference environment in reaction norm models is an inherent part of the population state, independent of the actual environment where the individuals develop. The state of the population thus determines which environment it is adapted to, that is, where the expected geometric mean fitness is maximized (the location of the adaptive peak or the growth rate peak along the environmental axes). The environment the population is adapted to is, in other words, an internal population property, independent of the external environment. It is, however, only when the external environment coincides with the internal reference environment, or vice versa, that the population is adapted to the current environment. This is formally a result of state‐space modeling theory, which is an important theoretical basis for quantitative genetics evolutionary modeling. As a consequence, the reference environments should be modeled as part of the evolutionary models (1) to (3). How this should be done is an open question, where the best answer may depend on the problem under study. One alternative is to let ***u***_ref_ be a function of an evolvable ***G*** matrix (e.g., Arnold, Bürger, Hohenlohe, Ajie, & Jones, 2008). That would give a complex solution, especially in the multivariate and nonlinear case, and this alternative is not further discussed (except in a simple numerical example in Section 2.2). As a straightforward solution, I propose that the reference environment vector may be modeled as a vector ${\overset{¯}{z}}_{c,t}$ of mean traits in their own right, just as other reaction norm traits. Equation (3) must accordingly be augmented with the ${\overset{¯}{z}}_{c,t}$ state variables. The details of this for parametrized models are developed in Section 2, while augmented function‐valued models are discussed in Appendix 1. Whether the reference traits in ${\overset{¯}{z}}_{c,t}$ are evolvable is also an open question, but considering the complexity of evolutionary processes, such evolvability cannot be excluded without good arguments. Also note that evolvable reference traits may be combined with an evolvable ***G*** matrix.

The idea of an evolvable reference trait was introduced in Ergon and Ergon (2017), but then based on biological arguments, as a result of the novel idea of a perception trait as a means of relaxing constraints on the evolution of reaction norms. A main purpose of the present article is to show that the plasticity reference environment not only *may* be modeled but that it in principle *must* be modeled, in one way or another, as part of the quantitative genetics state‐space model (although this is not necessary if the reference environment is not evolvable).

The reference environment vector ${\overset{¯}{z}}_{c,t}$ is closely related to the environment the population is adapted to, which we may denote ***u***_0_. As discussed in detail for the special case in Ergon and Ergon (2017), an unsymmetrical distribution of the phenotype ***y*** results in a difference between ${\overset{¯}{z}}_{c,t}$ and ***u***_0_, but at equilibrium in a stationary stochastic environment the expected deviation is independent of the mean values **μ**_*U*_ and **μ**_**Θ**_ of ***u***_*t*_ and **θ**_*t*_, respectively.

Under the assumption that some elements in the environmental reference trait vector are genetically variable, these elements must be included in the state equation (3), or its function‐valued counterpart. In Section 2, I show how this can be done for multivariate and nonlinear parametrized reaction norms. If all elements in the reference environment have zero genetic variance in the population, they can without consequence be set to zero, and this is thus an implicit assumption in traditional reaction norm models.

As discussed in Ergon and Ergon (2017), an important result of a fully evolvable plasticity reference environment is the property of complete genetic assimilation, by which “selection can act in such a manner as to turn an environmentally stimulated phenotype (i.e., plasticity) into a fixed response to prevalent environmental conditions (assimilation)” (Pigliucci & Murren, 2003). I here use the term “complete genetic assimilation” as in Ergon and Ergon (2017), to describe the evolutionary scenarios where, after an abrupt environmental change, there is an initial increase in phenotypic plasticity, after which the mean plasticity is reduced and the environment range, or value, to which the population is adapted moves toward the current mean environment. This entails that all elements in the reference environment vector have genetic variability, such that they are evolvable.

A major difficulty of the approach with evolvable reference traits is to find empirical measures of these latent parameters. In the linear and univariate example in Ergon and Ergon (2017), for example, individual reference traits *z*_*c*_ (horizontal reaction norm variation) cannot be distinguished from *z*_*a*_ (vertical reaction norm variation) by means of a static breeding design (Hill, 2010; Thompson, Brotherstone, & White, 2005). Different values of the variance *G*_*cc*_ of *z*_*c*_ will, however, give different dynamical responses to environmental variations, and assuming that the variance *G*_*aa*_ of *z*_*a*_ is known this can be used to identify *G*_*cc*_. It is also possible to identify several parameters (Appendix 2).

In Section 3, I simulate a set of linear reaction norms, to clarify why the environment *u*_0_ where the phenotypic variance has a minimum must be seen as a population characteristic. I also include a simulation example with multivariate and nonlinear reaction norms, where the environment changes in a sudden step, and where the property of complete genetic assimilation is demonstrated. In a third simulation example, I show the effect of evolvable environmental reference values on the results in Chevin and Lande (2015), regarding the plastic response in a population system with a single phenotype, and two correlated environmental cues. Finally, I include a discussion in Section 2.2.

In Appendix 1, I show that the plasticity reference environment needs to be modeled also in function‐valued models, and how that can be done for univariate and nonlinear reaction norms based on environmental index values. I also describe two additional problems in such cases.

In Appendix 2, I finally present a preliminary example showing how the variances of and covariances between quantitative reference traits may be identified from dynamical experiments.

An example Matlab code for the extended Chevin‐Lande simulation is provided in Data S1.

## 2 METHODS

### 2.1 Background state‐space theory

As a background and reference for the theoretical development, I include a summary of the underlying state‐space theory for discrete‐time systems. Caswell (2001, Ch. 3) refers to Zadeh's formal theory of state (Zadeh & Desoer, 1963), but state‐space modeling of dynamical systems is older than that. Of special historical importance is the seminal paper of Kalman (1960), concerning the discrete‐data linear filtering problem (Kalman filtering), although linear state‐space models are special cases.

The starting point for a general discrete‐time state‐space model is the idea of an abstract discrete‐time system that interacts with its environment through a vector **φ**_*t*_ of input variables and a vector ***y***_*t*_ of response variables. A vector ***x***_*t*_ of variables that takes its values in some set *X* (a state‐space) is a state vector if it satisfies the following two requirements:

1. There exists a function *g* (·) that uniquely determines the response at any time *t* as a function of the input and the state at *t*,
  (4) $$y_{t}=g\left(x_{t},\varphi_{t}\right).$$
2. There exists a function *f* (·) that uniquely determines the state at any time *t* as a function of the state at any earlier time *t*_0_ and the input sequence from *t*_0_ to *t *− 1, for any *t*_0_ and sequence **φ**_0_, **φ**_1_,…, **φ**_*t*−1_, that is, ***x***_*t*_ = *f* (*x*_0_, **φ**_0_, **φ**_1,_…, **φ**_*t*−1_). From this follows that ***x***_1_ = f(***x***_0_, **φ**_0_), and generally that ***x***_t_ at any time *t* can be propagated one step forward in time according to (Åström & Murray, 2008)
  (5) $$x_{t+1}=f\left(x_{t},\varphi_{t}\right).$$

The function *g* (·) is known as the output or observation function, and the function *f* (·) as the state function, while ***x***_*t*_ is the state. At *t *= *t*_0_ the state variables will have or be given some initial values, but from then on all information from the past is carried by the state variables. It should be noted that any current state may be the result of a large number of different initial states and input sequences, especially if *t*_0_ is far back in time, and the initial state cannot therefore be reconstructed from the current state without detailed knowledge of the entire input sequence. Also note that the excitation **φ**_*t*_ may be a combination of deterministic and stochastic signals, and that the functions *g* (·) and *f* (·) may include different parts of a common input vector **φ**_*t*_.

When *g* (·) and *f* (·) are linear, and when the stochastic part of **φ**_*t*_ is white (no autocorrelation) and normally distributed, the optimal mean value ${\overset{¯}{x}}_{t}$ can be found from ***y***_*t*_ using the Kalman filter, such that the covariance $E\left[\left(x_{t}-{\overset{¯}{x}}_{t}\right){\left(x_{t}-{\overset{¯}{x}}_{t}\right)}^{T}\right]$ is minimized (Lewis, Xie, & Popa, 2008; Newman et al., 2014). In the general case, estimates of the distribution of ***x***_*t*_ can be found from ***y***_*t*_ using the Chapman‐Kolmogorov equation and various techniques (Arulampalam, Maskell, Gordon, & Clapp, 2002; Newman et al., 2014).

### 2.2 Evolutionary state‐space models

Assuming sufficient genetic variation, the mean phenotypic values in a population will evolve when the environment varies from generation to generation. As summarized in the Introduction, mathematical modeling of this evolution for plastic organisms involves a state‐space model, which assuming non‐overlapping generations require three equations. First, equation (1) describes how a multivariate individual phenotype ***y***_*i,t*_ is expressed as a linear or nonlinear function of quantitative traits ***z***_0,*i*,*t*_ and a continuously varying developmental environment (cue vector) ***u***_*t*_. Second, equation (2) describes how the individual fitness depends on the difference between the phenotype ***y***_*i,t*_ and the vector **θ**_*t*_ of phenotypic expressions that maximizes fitness in the given generation. Third, the state equation may under given assumptions be the multivariate breeder's equation (3) (Lande, 1979).

**Figure 1:**
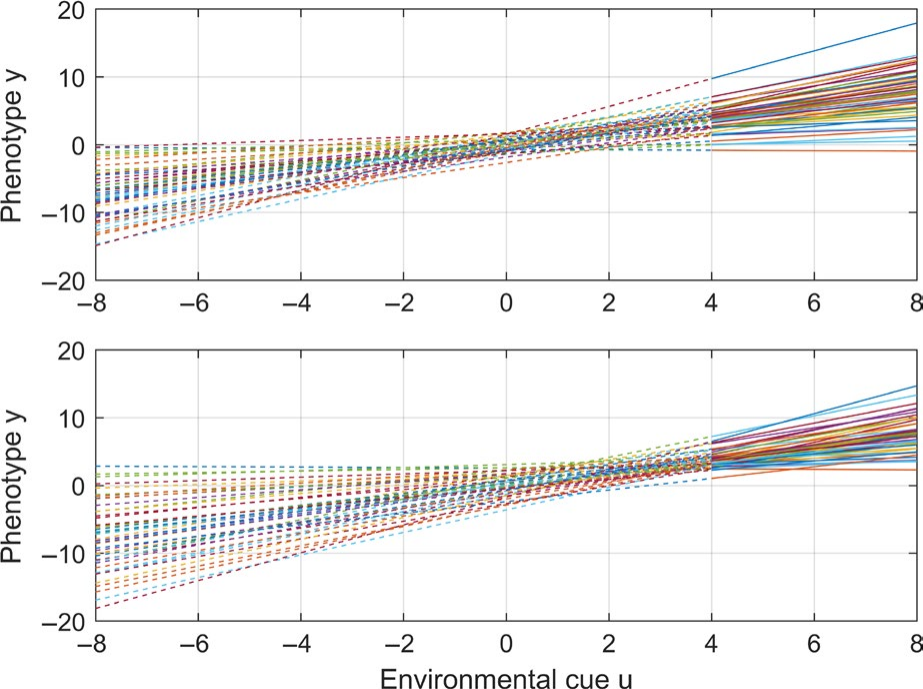
Two populations of reaction norms *y* = *a *+ *b* (*u* − *c*) + e, with $\overset{¯}{a}=0$, $\overset{¯}{b}=1$, *G*_*aa*_ = 0.25, *G*_*bb*_ = 0.2, $G_{\mathrm{ab}}=G_{\mathrm{ac}}=G_{\mathrm{bc}}=0$, and $\sigma_{e}^{2}=0.5$. The upper panel has $u_{0}=c=\overset{¯}{c}=u_{\mathrm{ref}}=0$ and *G*_*cc*_ = 0, which gives (with other parameter values) the two‐trait model of Lande (2009). The lower panel has $u_{0}=\overset{¯}{c}=2$ and *G*_*cc*_ = 0.25, which gives (with other parameter values) the three‐trait model of Ergon and Ergon (2017). Solid lines show the reaction norms in a limited range of current environmental values with mean μ_*U*_ = 6, that is, far away from the reference environment *u*_0_ = *u*_ref_ = 0 (upper panel) and $u_{0}=\overset{¯}{c}=2$ (lower panel). Dashed lines show extrapolations of the reaction norms, to emphasize that the cue value *u*_0_ where the phenotypic variance is minimized is a population characteristic, also if *u* varies in a range far from *u*_0_. If, in other words, the individual reaction norms are known only in a limited range of environments with a given mean value far from *u*_0_, the value of *u*_0_ will still be known. The lower panel indicates a higher value of the minimum phenotypic variance, owing to the *G*_*cc*_ > 0 value, that is, to the variance of the individual reaction norm positions along the environmental cue axis. Note that if μ_*U*_ would be hold constant forward in time, also *u*_0_ = 0 in the upper panel would stay constant, as in Lande (2009), while $u_{0}=\overset{¯}{c}$ in the lower panel would evolve toward μ_*U*_ = 6, as in Ergon and Ergon (2017)

When equation (1) is compared with the general state‐space output function (4), it is apparent that the environmental reference vector ***u***_ref_ must be part of either the current state or the current input. As equation (4) describes how the abstract discrete‐time system interacts with the current environment through the vector **φ**_*t*_ of input variables, and as a reference environment possibly far away from the current environment cannot be part of the current input, it must necessarily be an inherent part of the current state of the population (as illustrated in Figure 1 in Section 3). The current *individual state* is thus ${\left[z_{0,t}^{T}\;\;u_{\mathrm{ref}}^{T}\right]}^{T}$, which leaves ***u***_*t*_ as the current input in equation (4). Note, however, that also **θ**_*t*_ in the fitness function (2) is an input variable, such that the total current input is $\varphi_{t}={\left[\begin{array}{cc} u_{t}^{T} & \theta_{t}^{T} \end{array}\right]}^{T}$.

In traditional reaction norm models, the reference environment is assumed to be the same for all individuals in the population, and the current *mean state* is then ${\left[{\overset{¯}{z}}_{0,t}^{T}\;\;u_{\mathrm{ref}}^{T}\right]}^{T}$, that is, the reference environment is in principle a population state variable, although it is implicitly assumed be constant. The environment the population is adapted to, is, in other words, an internal population property, independent of the external environment. It is, however, only when the external environment coincides with the internal reference environment, or vice versa, that the population is adapted to the current environment. Again, note that ***u***_ref_ cannot be a part of the *current* input, which according to equation (4) interacts with the system. The state variable ***u***_ref_ thus determines which environment the population is adapted to, whether it coincides with the current environment or not.

Any population state variable must be modeled as a population mean value, a variance or a higher order statistical moment, or functions of the statistical moments. As we must assume that the population may be adapted to different stationary stochastic environments, independent of constant ***G*** and ***P*** matrices, and as the elements in ***u***_ref_ must have the same dimensions as the elements in ***u***_*t*_ (e.g., temperature and salinity), the remaining choice is a mean trait vector, which we may denote ${\overset{¯}{z}}_{c,t}$. Note that ***u***_ref_ should be modeled in this way also when it is set to zero, and that we in general must assume that ${\overset{¯}{z}}_{c,t}$ may be evolvable. As described in Section 2.2, this way of modeling a possibly varying input is natural also from an engineering control point of view. As mentioned in Section 1, we could alternatively let ***u***_*ref*_ be a function of an evolvable ***G*** matrix (e.g., Arnold et al., 2008), but that possibility is not discussed further in this article (except in a simple numerical example in the Section 2.2). As also mentioned in the Introduction, an evolvable ***G*** matrix may come in addition to an evolvable reference trait vector.

Setting $u_{\mathrm{ref}}={\overset{¯}{z}}_{c,t}$ raises the question of possible biological mechanisms for individual traits ***z***_*c*,*i*,*t*_. Ergon and Ergon (2017) proposed that individual reaction norms may be shifted along the cue axis according to how individuals perceive the environment, which results in individual perception traits. In the general multivariate and nonlinear case, such perception effects will lead to individual trait vectors ***z***_*c*,*i*,*t*_, that thus should replace ***u***_ref_ in equation (1). Assuming that ***z***_*c*,*i*,*t*_, just as ***z***_0,*i*,*t*_, can be split into two independent and multinormally distributed parts, ***z***_*c*,*i*,*t*_ = ***x***_*c*,*i*,*t*_ + ***e***_*c*,*i*,*t*_, and that the additive genetic covariance matrix $G_{\mathrm{cc}}=E\left[\begin{array}{c} \left(x_{c,i,t}-{\overset{¯}{x}}_{c,t}\right){\left(x_{c,i,t}-{\overset{¯}{x}}_{c,t}\right)}^{T} \end{array}\right]$ is positive definite, the mean traits in ${\overset{¯}{z}}_{c,t}$ will be evolvable. This results in a dynamical reference environment, which in a stationary stochastic environment will evolve into an equilibrium.

With ***u***_ref_ = ***z***_*c*,*i*,*t*_, the model (1, 2, 3) will according to the multivariate breeder's equation result in the augmented state‐space model

(6) $$y_{i,t}=g\left(z_{0,i,t},u_{t}-z_{c,i,t}\right)$$

(7) $$\begin{array}{cc} \left[\begin{array}{c} {\overset{¯}{z}}_{0,t+1}\\ {\overset{¯}{z}}_{c,t+1} \end{array}\right]= & \left[\begin{array}{c} {\overset{¯}{z}}_{0,t}\\ {\overset{¯}{z}}_{c,t} \end{array}\right]+\frac{1}{{\overset{¯}{W}}_{t}}G_{\mathrm{aug}}P_{\mathrm{aug}}^{-1}\left[\begin{array}{c} \mathrm{cov}\left(W_{i,t},z_{0,i,t}\right)\\ \mathrm{cov}\left(W_{i,t},z_{c,i,t}\right) \end{array}\right]\\ & =\left[\begin{array}{c} {\overset{¯}{z}}_{0,t}\\ {\overset{¯}{z}}_{c,t} \end{array}\right]+G_{\mathrm{aug}}\beta_{t}, \end{array}$$

where

$$G_{\mathrm{aug}}=\left[\begin{array}{cc} G & E\left[\left(x_{0,i,t}-{\overset{¯}{x}}_{0,t}\right){\left(x_{c,i,t}-{\overset{¯}{x}}_{c,t}\right)}^{T}\right]\\ E\left[\left(x_{c,i,t}-{\overset{¯}{x}}_{c,t}\right){\left(x_{0,i,t}-{\overset{¯}{x}}_{0,t}\right)}^{T}\right] & G_{\mathrm{cc}} \end{array}\right]$$

and

$$P_{\mathrm{aug}}=\left[\begin{array}{cc} P & E\left[\left(z_{0,i,t}-{\overset{¯}{z}}_{0,t}\right){\left(z_{c,i,t}-{\overset{¯}{z}}_{c,t}\right)}^{T}\right]\\ E\left[\left(z_{c,i,t}-{\overset{¯}{z}}_{c,t}\right){\left(z_{0,i,t}-{\overset{¯}{z}}_{0,t}\right)}^{T}\right] & E\left[\left(z_{c,i,t}-{\overset{¯}{z}}_{c,i,t}\right){\left(z_{c,t}-{\overset{¯}{z}}_{c,t}\right)}^{T}\right] \end{array}\right],$$

while **β**_*t*_ is the selection gradient. Here, ***G***_*cc*_ = 0 results in $x_{c,i,t}={\overset{¯}{x}}_{c,t}$, and thus a constant mean state variable ${\overset{¯}{z}}_{c,t+1}={\overset{¯}{z}}_{c,t}$. In that special case, we may without further consequences set ${\overset{¯}{z}}_{c,t}=z_{c,i,t}=u_{\mathrm{ref}}=0$. In case only some of the traits in ***z***_*c*,*i*,*t*_ have genetic variability, only such traits should be included in equation [Link], while the others may be set to zero. In equation [Link], *W*_*i*,*t*_ and ${\overset{¯}{W}}_{t}$ are still computed from equation (2). Evolution in a stationary stochastic environment will lead to an equilibrium, where $E\left[\text{cov}(W_{i,t},z_{0,i,t}\right]=0$ and $E\left[\text{cov}(W_{i,t},z_{c,i,t}\right]=0$, that is, where the expected selection gradient is $E\left[\beta_{t}\right]=0$.

### 2.3 Parametric reaction norm modeling

With ***z***_0,*i*,*t*_ split into elevation traits ***z***_*a*,*i*,*t*_ and slope and shape traits ***z***_*b*,*i*,*t*_, the reaction norm function in equation (7) becomes

(8) $$y_{i,t}=g\left(z_{a,i,t},z_{b,i,t},u_{t}-z_{c,i,t}\right)\;\;.$$

Following Gavrilets and Scheiner (1993a), this function can be approximated by a power series in terms of the components of *q* environmental cues, with *p* different products of *u*_1,*t*_ − *z*_*c*,1,*i*,*t*_, *u*_2,*t*_ − *z*_*c*,2,*i*,*t*_, …, *u*_*q*,*t*_ − *z*_*c*,*q*,*i*,*t*_, such as *u*_1,*t*_ − *z*_*c*,1,*i*,*t*_, (*u*_1,*t*_ −*z*_*c*,1,*i,t*_)^2^, (*u*_1,*t*_ − *z*_*c*,1,*i,t*_) (*u*_2,*t*_ − *z*_*c*,2,*i,t*_) etc. This yields the individual reaction norm equation

(9) $$y_{i,t}=z_{a,i,t}+Z_{b,i,t}{\tilde{u}}_{i,t},$$

where ${\tilde{u}}_{i,\;t}$ is a *p* × 1 vector of all the different cue products involved, as, for example, *u*_1,*t*_ − *z*_*c*,1,*i*,*t*_, (*u*_1,*t*_ − *z*_*c*,1,*i,t*_)^2^, (*u*_1,*t*_ − *z*_*c*,1,*i*,*t*_) (*u*_2,*t*_ − *z*_*c*,2,*i,t*_) etc. With *m* phenotypic variables, ***y***_*i*,*t*_ and ***z***_*a,i,t*_ are *m* × 1 vectors, and ***Z***_*b,i,t*_ an *m* × *p* matrix of individual quantitative traits (see multivariate and nonlinear simulation example in Section 3). The elements in ***Z***_*b*,*i*,*t*_ can be ordered in an individual vector ***z***_*b,i,t*_ in any chosen way. We may, for example, have ***z***_*b,i,t*_ = vec(***Z***_*b,i,t*_), where vec(***Z***_*b,i,t*_) is a vector form of ***Z***_*b*,*i*,*t*_ such that the columns are linked into a single column vector of length *m* × *p*. Note that all of ***z***_*a*,*i*,*t*_, ***z***_*b*,*i*,*t*_ and ***z***_*c*,*i*,*t*_ may have independent additive genetic and non‐additive parts. When equation (7) is replaced by equation [Link], equation [Link] must be replaced by

(10) $$\left[\begin{array}{c} {\overset{¯}{z}}_{a,t+1}\\ {\overset{¯}{z}}_{b,t+1}\\ {\overset{¯}{z}}_{c,t+1} \end{array}\right]=\left[\begin{array}{c} {\overset{¯}{z}}_{a,t}\\ {\overset{¯}{z}}_{b,t}\\ {\overset{¯}{z}}_{c,t} \end{array}\right]+\frac{1}{{\overset{¯}{W}}_{t}}G_{\mathrm{aug}}P_{\mathrm{aug}}^{-1}\left[\begin{array}{c} \mathrm{cov}\left(W_{i,t},z_{a,i,t}\right)\\ \mathrm{cov}\left(W_{i,t},z_{b,i,t}\right)\\ \mathrm{cov}\left(W_{i,t},z_{c,i,t}\right) \end{array}\right].$$

The total number of state variables is thus *m* + *m* × *p* + *q*, where *q* is the number of environmental cues.

Note that the system (9, 10) has the external references **μ**_**Θ**_, **μ**_*U*_, and cov(***U***,**Θ**) through the fitness function (2). It follows from Ergon and Ergon (2017) that a symmetric phenotypic distribution *p* (***y***) at equilibrium in a stationary stochastic environment results in $E\left[{\overset{¯}{z}}_{a,t}\right]=\mu_{\Theta }$ and $E\left[{\overset{¯}{z}}_{c,t}\right]=\mu_{U}$, while an unsymmetrical *p*(***y***) leads to deviations from **μ**_**Θ**_ and **μ**_***U***_. These deviations will, however, be independent of the actual values of **μ**_*U*_ and **μ**_**Θ**_, such that a positive definite matrix ***G***_*cc*_ gives complete genetic assimilation in any stationary stochastic environment. It also follows from Ergon and Ergon (2017) and McNamara et al. (2011), that the mean slope values around the origin in a stationary stochastic environment is a function of cov(***U***,Θ).

## 3 RESULTS

### 3.1 Adaptive peak as population characteristic

Theoretical and simulation results for a simple linear example system with an evolvable plasticity reference environment are discussed in detail in Ergon and Ergon (2017). Here, I take a closer look at the linear reaction norms in that example, to show why the environmental cue *u*_0_ where the phenotypic variance is minimized, that is, the location of the adaptive peak, is a population characteristic. In the example system simulated in Ergon and Ergon (2017), the linear three‐trait reaction norm essentially is (letting only the elevation trait *z*_*a*_ = *a* + *e* have a non‐additive component, while *z*_*b*_ = *b* and *z*_*c*_ = *c*)

(11) $$y=a+b\left(u-c\right)+e,$$

which with $\overset{¯}{c}=0$ and *G*_*cc*_ = 0 gives the two‐trait reaction norm in Lande (2009). As shown in Ergon and Ergon (2017), the environment where the phenotypic variance has a minimum is $u_{0}=\overset{¯}{c}+\left(\overset{¯}{b}G_{\mathrm{bc}}-G_{\mathrm{ab}}\right)/G_{\mathrm{bb}}$, that is, $u_{0}=\overset{¯}{c}$ for *G*_*bc*_ = *G*_*ab*_ = 0.This implies that *u*_0_ is a population property, which as shown in Figure 1 may be located far away from the current external environment.

### 3.2 A multivariate and nonlinear case

**Figure 2:**
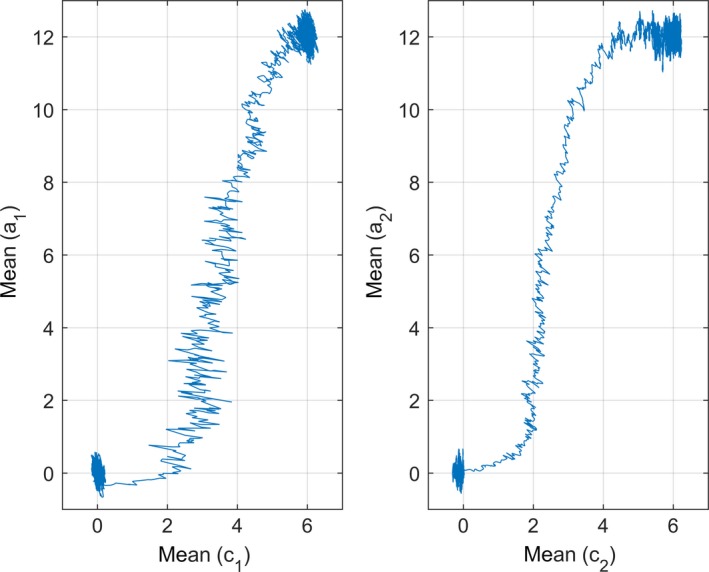
Step response phase portraits, that is, ${\overset{¯}{a}}_{1}=f\left({\overset{¯}{c}}_{1}\right)$ and ${\overset{¯}{a}}_{2}=f\left({\overset{¯}{c}}_{2}\right)$, for a system with the individual reaction norm model (12) and fitness functon (13), with steps in $\mu_{U_{1}}$ and $\mu_{U_{2}}$ from 0 to 6, and in $\mu_{\Theta_{1}}$ and $\mu_{\Theta_{2}}$ from 0 to 12, applied at *t *= 5,000 generations. The simulation ended at *t* = 10,000 generations. The ***G*** matrix was diagonal with $G_{a_{1}a_{1}}=G_{a_{2}a_{2}}=G_{c_{1}c_{1}}=G_{c_{2}c_{2}}=0.5$ and $G_{b_{11}b_{11}}=G_{b_{12}b_{12}}=G_{b_{22}b_{22}}=G_{b_{23}b_{23}}=0.045.$ The other parameters were $\sigma_{e_{1}}^{2}=\sigma_{e_{2}}^{2}=0.5$, $\sigma_{U_{1}}^{2}=\sigma_{U_{2}}^{2}=0.4$, $\text{cov}\left(u_{1},u_{2}\right)=0.2$, $\sigma_{\Theta_{1}}^{2}=\sigma_{\Theta_{2}}^{2}=1.6$, $\text{cov}\left(\theta_{1},\theta_{2}\right)=0.05$, $\text{cov}\left(u_{1},\theta_{1}\right)=\text{cov}\left(u_{2},\theta_{1}\right)=\text{cov}\left(u_{1},\theta_{2}\right)=\text{cov}\left(u_{2},\theta_{2}\right)=0.2$, and ω^2^ = 10

As also discussed in Ergon and Ergon (2017), as well as in the Introduction, an important consequence of an evolvable reference environment is complete genetic assimilation in any stationary environment. Here, I simulate a multivariate and nonlinear system, where complete genetic assimilation as defined in the Introduction takes place. Figure 2 shows step response phase portraits, that is, ${\overset{¯}{a}}_{1}=f\left({\overset{¯}{c}}_{1}\right)$ and ${\overset{¯}{a}}_{2}=f\left({\overset{¯}{c}}_{2}\right)$, for a system with the individual reaction norm model

(12) $$\left[\begin{array}{c} y_{1}\\ y_{2} \end{array}\right]=\left[\begin{array}{c} a_{1}\\ a_{2} \end{array}\right]+\left[\begin{array}{ccc} b_{11} & b_{12} & 0\\ 0 & b_{22} & b_{23} \end{array}\right]\left[\begin{array}{c} u_{1}-c_{1}\\ {\left(u_{1}-c_{1}\right)}^{2}\\ \left(u_{1}-c_{1}\right)\left(u_{2}-c_{2}\right) \end{array}\right]+\left[\begin{array}{c} e_{1}\\ e_{2} \end{array}\right],$$

with correlated cues *u*_1_ and *u*_2_, and with independent and zero mean white noise components *e*_1_ and *e*_2_. The fitness function was

(13) $$W=W_{\max}\exp\left(-{\left(y_{1}-\theta_{1}+y_{2}-\theta_{2}\right)}^{2}/2\omega^{2}\right),$$

with correlated values of θ_1_ and θ_2_. The state equation (8) was used, with $z_{a,i,t}={\left[\begin{array}{c} a_{1,i,t}+e_{1,i,t}\;\;\;a_{2,i,t}+e_{2,i,t} \end{array}\right]}^{T}$, $z_{b,i,t}={\left[\begin{array}{c} b_{11,i,t}\;\;\;b_{12,i,t}\;\;\;\begin{array}{c} b_{22,i,t}\;\;\;b_{23,i,t} \end{array} \end{array}\right]}^{T}$ and $z_{c,i,t}={\left[\begin{array}{c} c_{1,i,t}\;\;\;c_{2,i,t} \end{array}\right]}^{T}$. Note that the plots show that complete genetic assimilation takes place.

**Figure 3:**
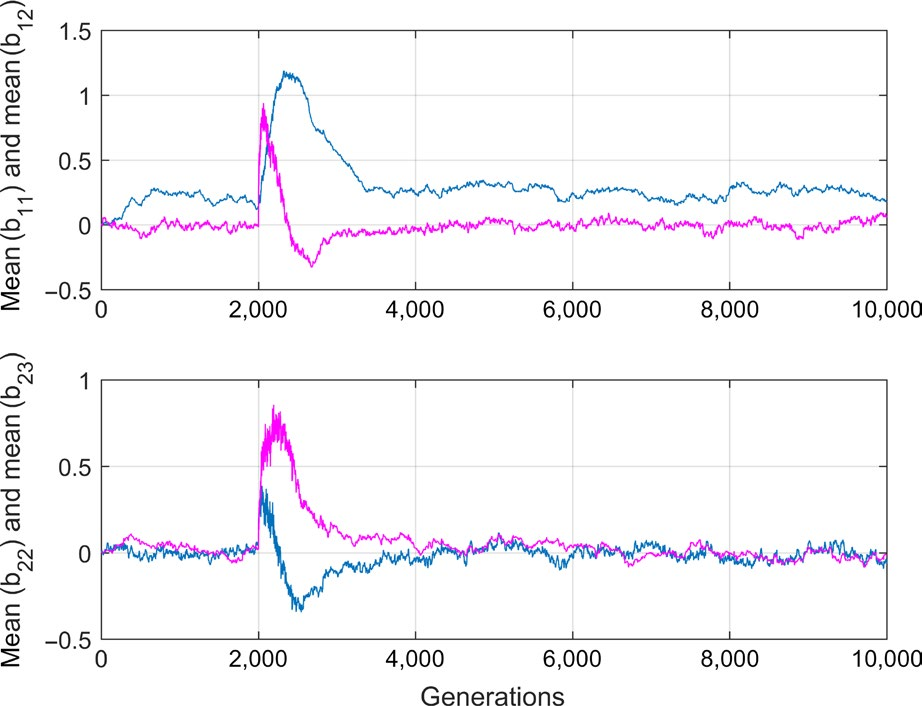
Mean plasticity slopes as function of time (generations) corresponding to the step response phase plots in Figure 2. Upper panel shows ${\overset{¯}{b}}_{11}$ (blue) and ${\overset{¯}{b}}_{12}$ (magenta), and lower panel shows ${\overset{¯}{b}}_{22}$ (blue) and ${\overset{¯}{b}}_{23}$ (magenta). All initial parameter values were set to zero

Figure 3 shows the corresponding mean plasticity slope plots. Note that only ${\overset{¯}{b}}_{11}$ is different from zero in a stationary stochastic environment, which may have implications for the possibilities to find parameter values from collected data (see Section 2.2 and Appendix 2).

### 3.3 Extended Chevin‐Lande example

More generally, evolvable reference environments will have profound effects on all types of evolutionary modeling involving reaction norms. Here, I show how it will affect the analysis of Chevin and Lande (2015), regarding how reaction norm slope values respond to correlated multiple environmental variables. Chevin and Lande studied the plastic response in a population system with a single phenotype and two environmental cues (environments of development) $u_{1,t}=\varepsilon_{1,d,t}$ and *u*_2,*t*_ = ɛ_2,*d*,*t*_, and the phenotypic expression that maximizes fitness $\theta_{t}=\varepsilon_{1,s,t}+\varepsilon_{2,s,t}$, where ɛ_1,*s*,*t*_ and ɛ_2,*s*,*t*_ are the environments of selection, and where $\mu_{\theta }=B\left(\mu_{U_{1}}+\mu_{U_{2}}\right)$. They used the traditional approach with reference environments equal to zero, that is, with an individual reaction norm model

(14) $$y=a+b_{1}u_{1}+b_{2}u_{2}+e,$$

where *e* is an independent residual component of variation. This is an extension of the single input example in Lande (2009). The variance of *e* was not stated, as it is not explicitly needed in the Chevin‐Lande simulation method. With negligible plasticity cost, the individual fitness function is given by

(15) $$W=W_{\max}\exp\left(-{\left(y-\theta \right)}^{2}/2\omega^{2}\right).$$

In a simulation, Chevin and Lande showed how the scaled mean reaction norm slopes ${\overset{¯}{b}}_{1}/B$ and ${\overset{¯}{b}}_{2}/B$ settles into different final values from equal initial values ${\overset{¯}{b}}_{1}/B={\overset{¯}{b}}_{2}/B=1$. The reason for the different final slope values is that the two cues are correlated, and also correlated with the phenotypic expression θ that maximizes fitness, and the main point in the paper was thus that interpretation of the reaction norm slopes must take these correlations into account.

I repeated the Chevin and Lande simulations using the individual reaction norm model

(16) $$y=a+b_{1}\left(u_{1}-c_{1}\right)+b_{2}\left(u_{2}-c_{2}\right)+e,$$

and the state equation (8). I used the same fitness function and the same small plasticity cost values as in Figure 1a in Chevin and Lande (2015) (who needed some plasticity cost to compute Φ in their equation (A4)). The difference from results with zero cost values was indeed negligible. I used the same *G*_*aa*_ and *G*_*bb*_ values as in Chevin and Lande (2015), and let the *c* traits be independent of the *a* and *b* traits. The *G*_*cc*_ matrix was diagonal. I used $\sigma_{e}^{2}=0.5$ in all simulations (as in Lande, 2009). See Data S1 for Matlab code.

**Figure 4:**
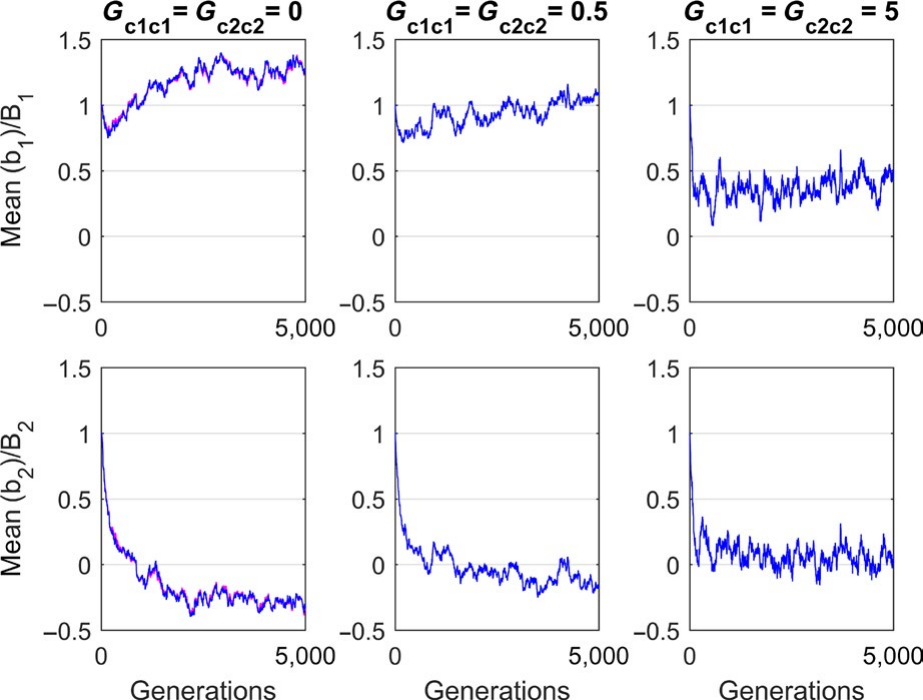
Evolution of scaled mean reaction norm slopes for the system in Equations (13), (14) and (8) in constant mean environments, from initial values equal to one to final stationary values ${\overset{¯}{b}}_{1,\infty }/B$ and ${\overset{¯}{b}}_{2,\infty }/B$ (blue). The ***G*** matrix was block‐diagonal with *G*_*aa*_ = 0.5, $G_{b_{1}b_{1}}=G_{b_{2}b_{2}}=0.04$, $G_{b_{1}b_{2}}=0.01$ and $G_{c_{1}c_{2}}=0$, while $G_{c_{1}c_{1}}$ and $G_{c_{2}c_{2}}$ varied. The residual variance was $\sigma_{e}^{2}=0.5$ in all simulations. Left panels show results for $G_{c_{1}c_{1}}=G_{c_{2}c_{2}}=0$, that is, for the case in Chevin and Lande (2015) (Figure 1a). The central and right panels show results for $G_{c_{1}c_{1}}=G_{c_{2}c_{2}}=0.5$ and $G_{c_{1}c_{1}}=G_{c_{2}c_{2}}=5$,respectively. For comparison, results using the Chevin‐Lande algorithm with $G_{c_{1}c_{1}}=G_{c_{2}c_{2}}=0$ are included in the left panels (magenta, not easy to see as it is overlapped by blue curve)

The simulation results in Figure 4 show that interpretation of the reaction norm slopes also must take the variances (and covariance) of the traits *c*_1_ and *c*_2_ into account. For $G_{c_{1}c_{1}}=G_{c_{2}c_{2}}=0$, the results for ${\overset{¯}{b}}_{1,t}/B$ and ${\overset{¯}{b}}_{2}/B\;$ are the same as in a simulation using the method in Chevin and Lande (2015) (Figure 1a). With increased values of $G_{c_{1}c_{1}}$ and $G_{c_{2}c_{2}}$, the final absolute values of ${\overset{¯}{b}}_{1,t}/B$ and ${\overset{¯}{b}}_{2,t}/B$ were reduced. Very large values of $G_{c_{1}c_{1}}$ and $G_{c_{2}c_{2}}$ gave ${\overset{¯}{b}}_{1,t}/B\to 0$ and ${\overset{¯}{b}}_{2,t}/B\to 0$ for *t* → ∞.

## 4 DISCUSSION

The main point in this article is that the plasticity reference environment ***u***_ref_ is a population characteristic, that ought to be modeled as such, and this is the case also if it is set to zero. Under the assumption of constant additive genetic and phenotypic covariance matrices, the remaining choice is to model ***u***_ref_ as a vector ${\overset{¯}{z}}_{c}$ of mean traits. The corresponding additive genetic covariance matrix ***G***_*cc*_ may be zero, and we may then set ***u***_ref_ = 0. However, if ***G***_*cc*_ ≠ 0, at least some of the “reference traits” will evolve in a changing environment, and they must then be included in the augmented state equation [Link].

One may ask why not the covariance matrices ***G*** and ***P*** also should be modeled and included as state variables in the augmented state equation [Link], and the answer is yes, in principle they should. In such cases, evolvability of these matrices cannot be based on individual selection, but on, for example, mutations. Here, however, I assume that ***G*** and ***P*** are constant and not evolvable, such that augmentation with these matrices is not necessary. See Arnold et al. (2008) for a review of empirical, analytical, and simulation studies of the ***G*** matrix, with a focus on its stability and evolution.

The biological mechanism behind evolvable “reference traits” may be that individuals perceive the environment differently, as discussed in Ergon and Ergon (2017), and we could accordingly introduce individual “perception traits” ***z***_*c*_. As shown, such perception traits may be used also in multivariate and nonlinear cases, leading to parametrized models according equations (2), [Link], and (8). As shown in Appendix 1, perception traits may be used also in models based on index environment phenotypes, which through interpolation leads to function‐valued models. In such models, however, ***G***_*cc*_ > 0 leads to non‐normal distributions, which is in conflict with the assumptions behind the multivariate breeder's equations [Link] and (8). Another added difficulty is that the individual state variable *z*_*c*,*i*,*t*_ does not fit into a function found through interpolation between phenotypic index traits *z*_1,*i*,*t*_ to *z*_*r*,*i*,*t*_. A similar problem in a life‐history trait setting is discussed in Irwin and Carter (2013).

The state‐space model (9,10) could have been formulated just as a generalization of the model in Ergon and Ergon (2017), based on biological arguments for perception traits. In addition to that, however, my intention has here been to show that, independent of these arguments, modeling of the reference environment is in principle necessary from a basic state‐space modeling point of view.

The most important result from a practical point of view is that population systems with a positive definite covariance matrix ***G***_*cc*_ obtain complete genetic assimilation in any stationary stochastic environment, as discussed in the Introduction. This means that the reaction norms at equilibrium after a change from one mean environment to another will be shifted to the new environment without any change in slope and shape. The adaptive peak, as determined by the state of the population, thus moves such that the population becomes adapted to the new environment. This movement is illustrated in a phase plane plot in Ergon and Ergon (2017), as well as in Figure 2. Long after the change in mean environment, the complete genetic assimilation will return the mean fitness to its original value, which is an essential difference from the partial genetic assimilation obtained in Lande (2009). More generally, the mean phase space position values ${\overset{¯}{z}}_{a}$ and ${\overset{¯}{z}}_{c}$ in equation (8) will evolve to new equilibrium values, while the mean slope and shape values ${\overset{¯}{z}}_{b}$ after a transient period will return to the original values. As a result, the dynamical responses to variations around the mean of a stationary stochastic environment, that is, around an adaptive peak, will be independent of the environmental location of the adaptive peak. This is demonstrated in Figures 2 and 3 in Section 3. In practice, however, complete genetic assimilation to any environment must be limited by biological constraints, plasticity costs etc.

As an alternative to the modeling of the reference environment as a vector ${\overset{¯}{z}}_{c}$ of mean traits, ***u***_ref_ could be modeled by means of elements in an evolvable ***G*** matrix. For the simple system in Lande (2009), which was the starting point for Ergon and Ergon (2017), the reference environment is *u*_ref_ = −*G*_*ab*_/*G*_*bb*_, where Lande made the choice *G*_*ab*_ = 0. When the mean environmental cue in that example was shifted from 0 to 10, while the peak of the fitness function was shifted from 0 to 20, the value of *u*_ref_ would evolve from 0 to 10 if *G*_*ab*_/*G*_*bb*_ evolved from 0 to 10. As $|G_{\mathrm{ab}}|\leq \sqrt{G_{\mathrm{aa}}G_{\mathrm{bb}}}$, this would without change in the value *G*_*aa*_ = 0.5 require a change in *G*_*bb*_ from 0.045 to equal to or less than 0.005, while a constant value *G*_*bb*_ = 0.045 would imply *G*_*aa*_ ≥ 4.5. It would in any case mean a system quite different from the original one. The dynamical properties would therefore not be the same in the new stationary environment, and therefore, we would not have complete genetic assimilation in the strict sense described above.

As mentioned in the Introduction, a main difficulty appears to be to find estimates of ***G***_*cc*_ from data. With linear reaction norms, it is theoretically impossible to find ***G***_*cc*_ from data collected at stationarity, but as discussed in Ergon and Ergon (2017), signs of ***G***_*cc*_ ≠ 0 will show up in transient situations. For the simple example in Ergon and Ergon (2017), it is in fact possible to find *G*_*cc*_ from dynamical experiments, as used in engineering control system identification (Appendix 2). A more general application of such methods on evolutionary problems is an interesting area for future research.

It is also interesting to note that there may exist mean reaction norm slope and shape parameters that are different from zero only in dynamical situations, as demonstrated in Figure 3. Although the corresponding individual parameters will be different from zero also in a stationary stochastic environment, this may make it difficult to find the corresponding covariance parameters from data collected at stationarity. In such cases, these parameters may possibly be found using dynamical identification experiments as introduced in Appendix 2.

Under the assumption that all individuals develop in the same environment, genetic assimilation leads to good tracking properties, and thus good adaptation to slowly changing environments. This may reduce the need for nonlinear reaction norms, and also the details of this is an interesting area for future research.

Finally note that modeling of a constant reference cue as an undriven discrete‐time integrator as in equations [Link] and (8), with ***G***_cc_ = 0, has an interesting parallel in engineering control applications. Such modeling of a constant system input is there used to achieve model based integral control through state feedback, which assures that the stationary control error is zero also if the constant input is unknown (Friedland, 1986). It also makes it possible to follow an input ramp function without an ever increasing error. The similar effects of the state‐space models (6, 7) and (9, 10), with ***G***_cc_ ≠ 0, is the genetic assimilation in any stationary stochastic environment, as described above, and good tracking properties when the environment changes slowly.

## CONFLICT OF INTEREST

None declared.

## AUTHOR CONTRIBUTION

Rolf Ergon is the sole author of this article.

## Figures and Tables

**Figure 5:**
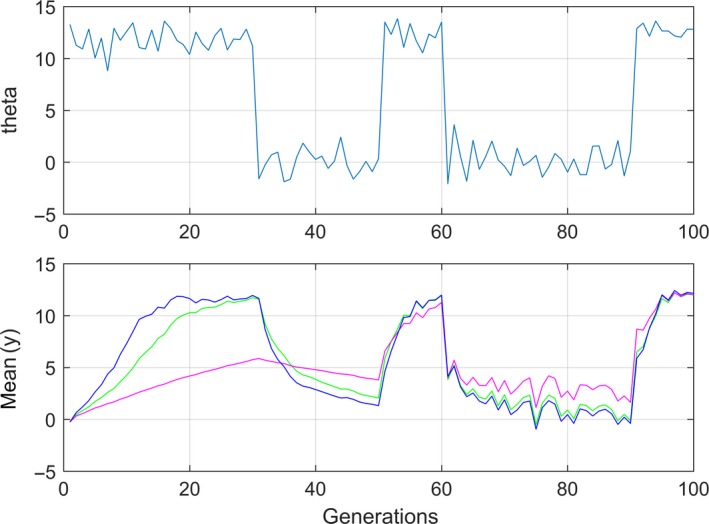
The input function θ_*t*_ = μ_Θ_ + *v*_θ,*t*_ (upper panel), and the model output for *G*_*cc*_ = 0 (magenta), *G*_*cc*_ = 0.5 (green) and *G*_*cc*_ = 1 (blue). The green curve is also the output from the assumed evolutionary system (A5) to (A7) itself

**Figure 6:**
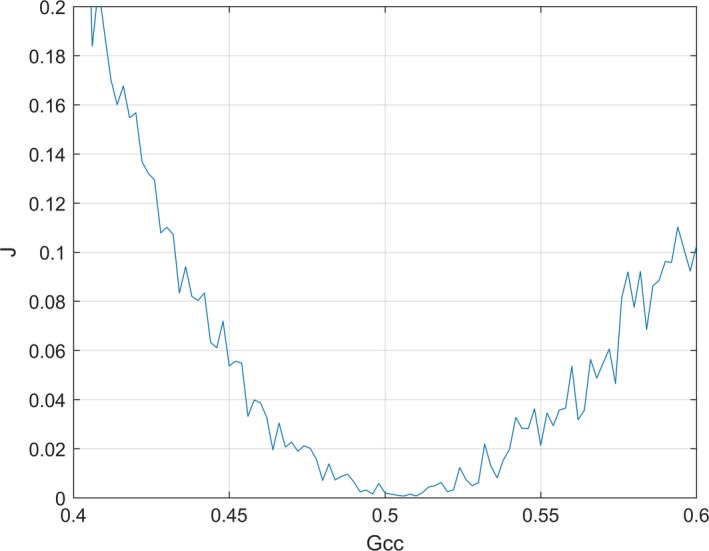
Identification of *G*_*cc*_ = 0.5 based on model with population size *N* = 10000

## APPENDIX 1

### Modeling based on index environment phenotypes

In order to show that the plasticity reference environment needs to be modeled also in function‐valued models, I here consider models based on index environment phenotypes. Such models lead to function‐valued models through interpolation between the index environments (Kingsolver et al., 2001; Kirkpatrick & Heckman, 1989). I also describe two additional problems in such cases. For clarity, I assume a univariate individual phenotype *y*_*i*,*t*_ and a univariate environmental cue *u*_*t*_.

In an index environment model, the phenotypic values ***y***_*i*,*t*_ in Equation (1) is defined as the individual phenotypic values at *r* discrete index environments,

(A1) $$y_{i,t}=\left[\begin{array}{c} y_{1,i,t}\\ \vdots \\ y_{r,i,t} \end{array}\right]=\left[\begin{array}{c} \gamma \left(u_{1,t}-z_{c,i,t}\right)\\ \vdots \\ \gamma \left(u_{r,t}-z_{c,i,t}\right) \end{array}\right],$$

where γ is the in general nonlinear reaction norm function, and where *z*_*c*,*i*,*t*_ is the individual reference trait (which is set to zero in traditional models). The individual phenotypic values are also used as individual traits, that is, *z*_1,*i*,*t*_ = *y*_1,*i*,*t*_ etc., and these traits have a phenotypic covariance matrix $P_{r}=\text{cov}\left(y_{i,t},y_{i,t}\right)$, and a corresponding additive genetic covariance matrix ***G***_*r*_. Setting *z*_*c*,*i*,*t*_ = 0, the multivariate breeder's Equation (3) would thus lead to

(A2) $$\left[\begin{array}{c} {\overset{¯}{z}}_{1,t+1}\\ \vdots \\ {\overset{¯}{z}}_{r,t+1} \end{array}\right]=\left[\begin{array}{c} {\overset{¯}{z}}_{1,t}\\ \vdots \\ {\overset{¯}{z}}_{r,t} \end{array}\right]+\frac{1}{{\overset{¯}{W}}_{t}}G_{r}P_{r}^{-1}\left[\begin{array}{c} \text{cov}\left(W_{i,t},y_{1,i,t}\right)\\ \vdots \\ \text{cov}\left(W_{i,t},y_{r,i,t}\right) \end{array}\right]$$

When *z*_*c*,*i*,*t*_ ≠ 0, this state equation must be augmented into

(A3) $$\left[\begin{array}{c} {\overset{¯}{z}}_{1,t+1}\\ \vdots \\ \begin{array}{c} {\overset{¯}{z}}_{r,t+1}\\ {\overset{¯}{z}}_{c,t+1} \end{array} \end{array}\right]=\left[\begin{array}{c} {\overset{¯}{z}}_{1,t}\\ \vdots \\ \begin{array}{c} {\overset{¯}{z}}_{r,t}\\ {\overset{¯}{z}}_{c,t} \end{array} \end{array}\right]+\frac{1}{{\overset{¯}{W}}_{t}}G_{\mathrm{rc}}P_{\mathrm{rc}}^{-1}\left[\begin{array}{c} \text{cov}\left(W_{i,t},y_{1,i,t}\right)\\ \vdots \\ \begin{array}{c} \text{cov}\left(W_{i,t},y_{r,i,t}\right)\\ \text{cov}\left(W_{i,t},z_{c,i,t}\right) \end{array} \end{array}\right],$$

where ***G***_*rc*_ and ***P***_*rc*_ are the covariance matrices of the vector ***y***_*i*,*t*_ augmented with *z*_*c*,*i*,*t*_. This raises two problems. First, with *z*_*c*,*i*,*t*_ ≠ 0 the traits $z_{1,i,t}=y_{1,i,t}$ etc. will not be normally distributed, even if the reaction norm has underlying normally distributed parameters, which is in conflict with the assumptions behind the multivariate breeder's equation (Lande, 1979). Equation (A3) will therefore be more of an approximation than it otherwise would be. Second, the state variable *z*_*c*,*i*,*t*_ does not fit into a function found through interpolation between *z*_1,*i*,*t*_ to *z*_*r*,*i*,*t*_. A similar problem in a life‐history trait setting is discussed in Irwin and Carter (2013). A possible solution is to assume that *z*_*c*,*i*,*t*_ is independent of *z*_1,*i*,*t*_ to *z*_*r*,*i*,*t*_, and model the evolution of ${\overset{¯}{z}}_{c,t}$ independently, that is, to use Equation (A2) combined with

(A4) $${\overset{¯}{z}}_{c,t+1}={\overset{¯}{z}}_{c,t}+\frac{1}{{\overset{¯}{W}}_{t}}G_{\mathrm{cc}}P_{\mathrm{cc}}^{-1}\text{cov}\left(W_{i,t},z_{c,i,t}\right).$$

## APPENDIX 2

### Preliminary example of evolutionary system identification

System identification is a mature discipline in the engineering control community, with prediction error methods developed during the 1980's (Ljung 1999), and subspace methods from the 1990's and later (Qin 2006). For evolutionary system identification, predictor error methods of the output error (OE) type is a straightforward choice.

Here is an example of the OE prediction error method applied on an evolutionary system identification problem. Assume a system essentially as in Ergon and Ergon (2017), with the individual reaction norm

(A5) $$y=a+b\left(u-c\right)+e,$$

the individual fitness function

(A6) $$W=\exp\left(-\frac{{\left(y-\theta \;\right)}^{2}}{2\omega^{2}}\right),$$

and the multivariate breeder's equation

(A7) $$\left[\begin{array}{c} {\overset{¯}{z}}_{a,t+1}\\ {\overset{¯}{z}}_{b,t+1}\\ {\overset{¯}{z}}_{c,t+1} \end{array}\right]=\left[\begin{array}{c} {\overset{¯}{z}}_{a,t}\\ {\overset{¯}{z}}_{b,t}\\ {\overset{¯}{z}}_{c,t} \end{array}\right]+\frac{1}{{\overset{¯}{W}}_{t}}{GP}^{-1}\left[\begin{array}{c} cov(W_{i,t},z_{a,i,t})\\ cov(W_{i,t},z_{b,i,t})\\ cov\left(W_{i,t},z_{c,i,t}\right) \end{array}\right],$$

where *z*_*a*_ = *a* + *e*,* z*_*b*_ = *b* and *z*_*c*_ = *c*. Here, *u* is the environmental cue, while θ is the phenotypic value that maximizes fitness. Assume ω = 10, and

$$G=\left[\begin{array}{ccc} G_{\mathrm{aa}} & 0 & 0\\ 0 & G_{\mathrm{bb}} & 0\\ 0 & 0 & G_{\mathrm{cc}} \end{array}\right]=\left[\begin{array}{ccc} 0.5 & 0 & 0\\ 0 & 0.045 & 0\\ 0 & 0 & 0.5 \end{array}\right],$$

and

$$P=\left[\begin{array}{ccc} P_{\mathrm{aa}} & 0 & 0\\ 0 & P_{\mathrm{bb}} & 0\\ 0 & 0 & P_{\mathrm{cc}} \end{array}\right]=\left[\begin{array}{ccc} 1 & 0 & 0\\ 0 & 0.045 & 0\\ 0 & 0 & 0.5 \end{array}\right].$$

Also assume θ_*t*_ = μ_Θ_ + *v*_θ,*t*_ as shown in Figure A1, upper panel, where μ_θ_ is piecewise constant, while *v*_θ,*t*_ is white noise with variance $\sigma_{v_{\theta ,t}}^{2}=1.6$. Assume *u*_*t*_ = μ_*u*_ + *v*_*u*,*t*_, where also *v*_*u*,*t*_ is white noise with variance $\sigma_{v_{u,t}}^{2}=0.4$, and where μ_*U*_ = 0.5μ_*Θ*_, and let θ_*t*_ and *u*_*t*_ be correlated, with $cov\left(\theta_{t},u_{t}\right)=0.2.$ Inputs like μ_*Θ*_ and μ_*U*_ in Figure A1 can formally be generated as pseudo‐random binary signals (PRBS), which are often used for identification of engineering control systems (Ljung 1999).

Apply the input sequences θ_*t*_ and $u_{t}\;$on the evolutionary system (A5) to (A7), and collect the mean phenotype ${\overset{¯}{y}}_{t}$ for t=1 to *T*.

Now assume that *G*_*cc*_ is the only unknown parameter in the system (A5) to (A7). In order to find *G*_*cc*_, apply the input sequences θ_*t*_ and $u_{t}\;$on a system model with different values of *G*_*cc*_, and collect the resulting outputs ${\hat{\overset{¯}{y}}}_{t}$. Also compute the prediction error $\varepsilon_{t}=\overset{¯}{y}-{\hat{\overset{¯}{y}}}_{t}$ for each value of *G*_*cc*_. Results for three values of *G*_*cc*_ are shown in Figure A1, lower panel.

We may search for the value of *G*_*cc*_ that minimizes the quadratic criterion function $J=\frac{1}{T}\sum_{t=1}^{T}\varepsilon_{t}^{2}$. Results for 100 values of *G*_*cc*_ from 0.402 to 0.600 with population size *N* = 10,000 are shown in Figure A2. Smaller population sizes increase the noise in this plot significantly.

For identification of several unknown parameters, a better search method is needed. This requires experimental data that are informative enough, but it also requires a theoretical identifiability analysis (it may not be theoretically possible to identify all parameters). Also note that we must assume a model, i.e., a linear or nonlinear reaction norm, a fitness function, and a covariance structure.

The applicability of dynamical system identification methods in an evolutionary setting remains to be investigated.
