# Supplementary material for: The environmental zero‐point problem in evolutionary reaction norm modeling
Source: Ecol Evol. 2018 Mar 25;8(8):4031–41. doi: 10.1002/ece3.3929 (PMC5916289; doi:10.1002/ece3.3929)
Supplement: Supplementary file 1 [file ECE3-8-4031-s001.docx]

# Supporting Information to

The environmental zero-point problem

in evolutionary reaction norm modeling

Rolf Ergon

[rolf.ergon@usn.no](mailto:rolf.ergon@usn.no)

University College of Southeast Norway

Manuscript for Ecology and Evolution

January 10, 2018

**Matlab code for extended Chevin-Lande simulation example**

%% Extended Chevin-Lande Example

clear

N=10000 % Population size

T=5000 % Simulation time steps

vare=0.5;

w2=39.5; % Width of fitness function RE

w2t=w2+vare; % Width of fitness function C-L

Psia=[10000 5000 ; 5000 10000]; % Approximately zero plasticity cost

Psib=[40 20 ; 20 40]; % Higher plasticity cost

Psi=Psia; % Choice of plasticity cost

invPsi=inv(Psi);

K=w2t*invPsi;

for Case=1:3

%% Plot number

if Case==1 Gcc=[0 0 ; 0 0];

end

if Case==2 Gcc=[0.5 0 ; 0 0.5];

end

if Case==3 Gcc=[5 0 ; 0 5];

end

%% Chevin and Lande simulation

% Parameter values

G=[0.5 0 0 ; 0 0.04 0.01 ; 0 0.01 0.04];

L=[0 0 0 ; 0 K(1,1) K(1,2) ; 0 K(2,1) K(2,2)];

C=[4 2 ; 2 4];

D=[3 1.5 ; 0.5 1];

% Generate environmental data

y=(C(1,2)*D(1,1)-C(1,1)*D(2,1))/(C(1,2)^2-C(1,1)*C(2,2));

x=(D(1,1)-C(1,2)*y)/C(1,1);

w=(C(1,2)*D(1,2)-C(1,1)*D(2,2))/(C(1,2)^2-C(1,1)*C(2,2));

z=(D(1,2)-C(1,2)*w)/C(1,1);

p=sqrt(C(1,1)-x^2*C(1,1)-2*x*y*C(1,2)-y^2*C(2,2));

q=(C(1,2)-x*z*C(1,1)-x*w*C(1,2)-y*z*C(1,2)-y*w*C(2,2))/p;

r=sqrt(C(2,2)-z^2*C(1,1)-w^2*C(2,2)-2*z*w*C(1,2)-q^2);

randn1=randn(T,1);

randn2=randn(T,1);

randn3=randn(T,1);

randn4=randn(T,1);

for i=1:T

epsd1(i,1)=sqrt(C(1,1))*randn1(i,1);

epsd2(i,1)=(C(1,2)/C(1,1))*epsd1(i,1)+sqrt(C(2,2)-C(1,2)^2/C(1,1))*randn2(i,1);

epss10(i,1)=x*epsd1(i,1)+y*epsd2(i,1);

epss20(i,1)=z*epsd1(i,1)+w*epsd2(i,1);

epss1(i,1)=epss10(i,1)+p*randn3(i,1);

epss2(i,1)=epss20(i,1)+q*randn3(i,1)+r*randn4(i,1);

end

% Simulate and plot

if Case==1

g=[0 1 1]';

for i=1:T

b1(i)=g(2);

b2(i)=g(3);

invPhi=([1 epsd1(i,1) epsd2(i,1)]'*[1 epsd1(i,1) epsd2(i,1)]+L)/w2t;

Phi=inv(invPhi);

Theta=[epss1(i,1)+epss2(i,1) 0 0]';

g=g-G*inv(Phi+G)*(g-Theta);

end

figure(1)

subplot(2,3,Case),plot(b1,'m'),hold on

subplot(2,3,Case+3 ),plot(b2,'m'),hold on

xlabel('Generations')

axis([0 T -0.5 1.5])

end

%% Rolf Ergon simulation

u1=epsd1';

u2=epsd2';

theta=epss1'+epss2';

% Generate individual population traits around abar, bbar and cbar

z=sqrt(0.04-0.04/16);

for t=1:T

v=randn(N,1);

a(:,t)=sqrt(0.5)*randn(N,1);

bre1(:,t)=sqrt(0.04)*v;

bre2(:,t)=0.25*sqrt(0.04)*v+z*randn(N,1);

c1(:,t)=sqrt(Gcc(1,1))*randn(N,1);

c2(:,t)=sqrt(Gcc(2,2))*randn(N,1);

end

% Simulate and plot

abar=0;

b1bar=1;

b2bar=1;

c1bar=0;

c2bar=0;

Wbar=1;

abarplot=zeros(1,T);

b1barplot=zeros(1,T);

b2barplot=zeros(1,T);

c1barplot=zeros(1,T);

c2barplot=zeros(1,T);

Wbarplot=zeros(1,T);

cost=zeros(N,1);

for t=1:T

abarplot(t)=abar;

b1barplot(t)=b1bar;

b2barplot(t)=b2bar;

c1barplot(t)=c1bar;

c2barplot(t)=c2bar;

Wbarplot(t)=Wbar;

b1=b1bar+bre1(:,t);

b2=b2bar+bre2(:,t);

buc1=b1.*(u1(t)-c1bar-c1(:,t));

buc2=b2.*(u2(t)-c2bar-c2(:,t));

cost1=(b1bar+bre1(:,t))*invPsi(1,1)+(b2bar+bre2(:,t))*invPsi(2,1);

cost2=(b1bar+bre1(:,t))*invPsi(1,2)+(b2bar+bre2(:,t))*invPsi(2,2);

cost=cost1.*(b1bar+bre1(:,t))/2+cost2.*(b2bar+bre2(:,t))/2;

W=exp(-(abar+a(:,t)+buc1+buc2+sqrt(vare)*randn-theta(t)).^2/(2*w2)-cost);

Wbar=mean(W);

covparW=cov([a(:,t),bre1(:,t),bre2(:,t),c1(:,t),c2(:,t),W]);

abar=abar+0.5*covparW(6,1)/Wbar;

b1bar=b1bar+covparW(6,2)/Wbar; % Gaug*inv(Paug)=diag([0.5 1 1 1 1]),

b2bar=b2bar+covparW(6,3)/Wbar; % because Gaug and Paug are

c1bar=c1bar+covparW(6,4)/Wbar; % block-diagonal, with

c2bar=c2bar+covparW(6,5)/Wbar; % Pbb=Gbb and Pcc=Gcc

end

figure(1)

subplot(2,3,Case),plot(b1barplot,'b'),grid,hold off

if Case==1 title('G_c_1_c_1= G_c_2_c_2= 0')

end

if Case==2 title('G_c_1_c_1= G_c_2_c_2= 0.5')

end

if Case==3 title('G_c_1_c_1 = G_c_2_c_2 = 5')

end

if Case==1 ylabel('mean(b_1)/B_1')

end

axis([0 T -0.5 1.5])

subplot(2,3,Case+3 ),plot(b2barplot,'b'),grid,hold off

if Case==1 ylabel('mean(b_2)/B_2')

end

xlabel('Generations')

axis([0 T -0.5 1.5])

end
